# A Personalized Approach for Oligometastatic Prostate Cancer: Current Understanding and Future Directions

**DOI:** 10.3390/cancers17010147

**Published:** 2025-01-05

**Authors:** Parissa Alerasool, Susu Zhou, Eric Miller, Jonathan Anker, Brandon Tsao, Natasha Kyprianou, Che-Kai Tsao

**Affiliations:** 1 Department of Hematology-Oncology, Icahn School of Medicine at Mount Sinai, New York, NY 10029, USAche-kai.tsao@mssm.edu (C.-K.T.); 2 Department of Medicine, Montefiore Medical Center Albert Einstein College of Medicine, Bronx, NY 10467, USA; 3 Department of Medicine, Icahn School of Medicine at Mount Sinai (Morningside/West), New York, NY 10025, USA; 4 Department of Urology, Icahn School of Medicine at Mount Sinai, New York, NY 10029, USA

**Keywords:** prostate cancer, oligometastatic disease, oligometastasis, stereotactic body radiotherapy, focal therapy, androgen deprivation therapy, metastasis-directed therapy, castration-resistant prostate cancer, PSMA PET scan, radical prostatectomy

## Abstract

Oligometastatic prostate cancer (OMPC) represents an intermediate state in the progression from localized disease to widespread metastasis when the radiographically significant sites are limited in number and location. With no clear consensus on a definition, its diagnostic significance and associated optimal therapeutic approach remain controversial, posing a significant challenge for clinicians. The current standard of care for metastatic disease is to start systemic therapy; however, active surveillance and targeted radiotherapy have become attractive options to mitigate the long-term effects of androgen deprivation therapy (ADT). Furthermore, evolving biomarker methodologies may further define optimal treatment selection. In this review, we summarize the current understanding that guides the treatment of OMPC, with a focus on how host response can be an important contributing factor. Evolving scientific understanding and clinical development will continue to shape the landscape of treatment strategies for this distinct disease state.

## Simple Summary

Prostate cancer is the most common non-skin cancer in American men. In the progression from localized to metastatic prostate cancer, there lies an intermediate state of disease known as oligometastatic prostate cancer at which point cure may be possible. Defining oligometastasis and determining a standardized therapeutic approach has remained a challenge for clinicians. The aim of this study is to provide a comprehensive review of the current literature on this disease state in order to summarize current diagnostic and therapeutic approaches while paving the way for future developments. We found that a multifaceted approach either by intensifying or de-intensifying therapy with a combination of targeted and systemic therapies may be beneficial in patients with oligometastatic disease, although further clinical validation is needed.

## 1. Introduction

Prostate cancer is the most common non-skin cancer and the second most common cause of cancer-related death in American men [1,2]. Advances in diagnostic imaging with higher sensitivity for metastatic disease have increased the detection of OMPC, resulting in stage migration. Conventional imaging methods, such as computed tomography (CT) and magnetic resonance imaging (MRI), have relatively lower diagnostic sensitivity (42%) and specificity (82%) in patients with biochemically recurrent disease (BCD) [3]. Contemporary development of Prostate Specific Membrane Antigen (PSMA) targeting Positron Emission Tomography (PET) imaging has shown significant promise, although radiographic detection is highly dependent on PSA levels and kinetics [4].

Oligometastasis is a clinically significant state proposed by Hellman and Weichselbaum in 1995, in which certain tumors are limited by their anatomy and physiology to developing metastases concentrated in only a single or a limited number of organs [5]. The oligometastasis paradigm has been applied to a variety of malignancies, including lung, colorectal, renal, and prostate [6]. Further studies have established a molecular basis of oligometastasis and a state predictive of favorable clinical outcomes [7]. At the molecular level, oligometastatic cells differ from multi-metastatic cells, as they lack the ability to travel to “further” organs in the body and rather have a preferential tropism for “local” organs. Cells with the oligometastatic phenotype lack the ability to evade the body’s immunologic surveillance of cancer and thus fail to replicate indefinitely like multi-metastatic cells [8].

Oligometastatic (OM) disease can be further subtyped into “synchronous” or “metachronous”, as defined by the time between the primary diagnosis of cancer versus the development of oligometastases [9]. A subset of prostate cancer patients are found to have OM synchronous disease, in which the metastatic lesions are present at the time of initial diagnosis [10]. Metachronous (or oligorecurrent) disease, on the other hand, is when metastatic disease develops later (3 months or more) after initial treatment of the localized malignancy with curative intent [9]. Currently, goals of care and overall approach may differ depending on whether a patient has synchronous or metachronous disease, as well as site(s) of oligometastatic involvement. In patients with synchronous OM disease, often a combination of treatment for the primary prostate tumor and metastasis-directed therapy (MDT) is administered concurrently with a fixed duration of systemic therapy, potentially with “curative intent”. Conversely, with metachronous OM disease, “palliative” MDT with radiation or surgical resection targeting the individual sites of active disease, with or without indefinite systemic therapy, is more common [11]. Retrospective evidence from the Systemic Therapy in Advancing or Metastatic Prostate Cancer: Evaluation of Drug Efficacy (STAMPEDE) trial may serve as proof of concept that primary prostate cancer radiotherapy in combination with systemic therapy can lead to significantly improved overall survival and failure-free survival in patients with low metastatic burden, although controversy still surrounds this treatment approach [12,13]. Current challenges in the treatment of OMPC include a lack of definition consensus, absence of predictive biomarkers to direct therapy, an unknown role for prostate and metastasis-directed therapy, and no standardized systemic therapeutic approach for those with lower disease volume.

Several studies have enabled defining the disease state based on the number and the location of metastatic sites [14,15,16]. The presence of five or fewer extrapelvic metastatic lesions without visceral involvement can be classified as OMPC. Metastasis to the lung, liver, and brain is associated with a poorer prognosis, and the current consensus is that such clinical phenotypes should be treated aggressively with systemic therapy. Conversely, the optimal treatment for those with OMPC is less clear, and clinical development combining systemic therapy with prostate and metastasis-directed therapy is rapidly evolving [17,18,19,20,21]. We will discuss the evolving definition and therapeutic development of OMPC with a focus on the potential role of artificial intelligence in this disease state.

## 2. Biologic and Molecular Basis of OMPC

In an oligometastatic–polymetastatic xenograft model, microRNA 200-c enhancement in oligometastatic cell lines led to the progression of polymetastatic disease [22]. Such preclinical study provides proof of the principle that OMPC behaves differently than polymetastatic disease and distinct treatment strategies may be needed to achieve the maximal clinical benefit in patients by optimizing treatment efficacy and mitigating side effects.

**Figure 1:**
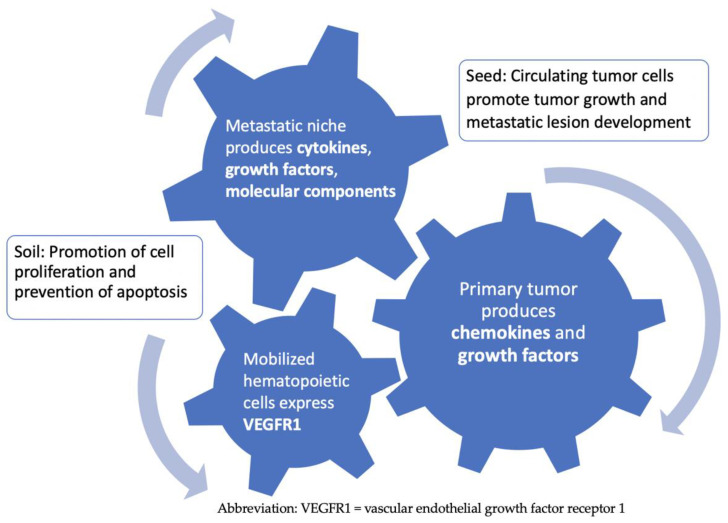
Metastatic niche and the seed and soil hypothesis of metastasis.

Steven Paget proposed the “Seed and Soil” hypothesis for metastasis, in which a receptive microenvironment is necessary for malignant cells to successfully travel to distant sites and develop into metastatic lesions [23]. Additional evidence suggests that the primary tumor can produce chemokines and growth factors that facilitate the microenvironment in certain distant tissues to support the development of metastatic disease [24]. The parenchyma of specific distant tissue is believed to undergo significant changes that allow for the development of metastatic lesions from tumor cells that have extravasated and invaded these sites [25]. The microenvironmental landscape of the metastatic niche provides growth factor signaling and cellular and molecular components like fibronectin that promote cell proliferation and suppress apoptosis [25]. Growth factors produced by the primary tumor lead to the mobilization of hematopoietic progenitor cells that express vascular endothelial growth factor receptor 1, thus creating the “premetastatic niche” and serving as the blueprint for the pattern of spread of metastatic disease [25] (Figure 1). Evidence from experimental models in melanoma, breast, and colon cancer supports the phenomenon of tumor self-seeding, in which circulating tumor cells (CTCs) are produced by the tumor itself, metastatic lesions, or a separate tumor mass [26]. These CTCs harbor continuous tumor growth and the development of metastatic sites through re-infiltration and self-seeding of the tumor mass [27].

Lineage plasticity is the ability of a tumor cell to transform from one developmental pathway to another pathway of differentiation through a combination of genomic and epigenetic events [28,29]. Recent advances in phenotypic landscape profiling have shown that the conversion of epithelial cells to mesenchymal cells involves profound phenotypic changes, including loss of cell–cell adhesion and polarity and acquisition of invasive properties [30,31,32]. The process of epithelial–mesenchymal transition (EMT) creates an opportunity for epithelial-derived tumors to become invasive and metastasize [30,31,32]. Indeed, EMT endows cells with migratory and invasive properties, induces stem cell properties, prevents apoptosis, and initiates metastasis. Loss of epithelial-cell markers and gain of mesenchymal-cell markers at the invasive front of solid tumors are linked to metastatic progression [30,31,32].

## 3. Assessing OMPC

Currently, radiographic lesion location and enumeration must be used to distinguish between the oligometastatic and polymetastatic disease states. Limited data are available to characterize the significance of genomic mutations, epigenetic modifications, and immunological pathways, which may distinguish different phenotypes. Recent evidence suggests that driver mutations like TP53 and WNT can predict patterns of metastatic spread of disease [33]. Deek et al. found patients with hormone-sensitive metastatic prostate cancer that harbors genomic aberration in TP53, WNT, DNA double-strand break (DDSB) repair, and cell-cycle pathways are associated with an increasing number of metastatic lesions [34]. Furthermore, in men with OMPC, TP53 mutation was associated with a shorter time to CRPC and shorter radiographic progression-free survival [34].

### 3.1. Circulating Tumor Cells (CTCs)

CTCs are cancer cells that have been released from the primary tumor into the bloodstream. Mandel et al. found that higher CTC levels in OMPC patients undergoing radical prostatectomy (RP) were associated with worse oncological outcomes (CTC count of ≥2 per 7.5 mL before or 6 months after surgery) [35]. Given the consistency in CTC levels at various time points pre- and post-surgery, these findings suggest that CTC monitoring may assist in determining which OMPC patients would benefit from radical prostatectomy [35].

### 3.2. PSMA-Based Imaging

Prostate-specific membrane antigen (PSMA) is overexpressed in 95% of prostate cancer cells, making PSMA-based radioligands a prime target for diagnostic development. In BCD, the detection of metastasis using clinical outcome correlation with Gallium(Ga)-68-PSMA PET had a per-patient sensitivity and specificity of 0.77 (95% confidence interval [95% CI], 0.46–0.93) and 0.97 (95% CI, 0.91–0.99), respectively [3]. In another study utilizing histopathologic confirmation, 68Ga-PSMA-11 PET was found to have a mean sensitivity and mean specificity of 0.74 (95% CI, 0.51–0.89) and 0.96 (95% CI, 0.85–0.99), respectively, at initial staging, and a mean positive predictive value of 0.99 (95% CI, 0.96–1.00) at BCD [36]. These findings underscore the significant value of PSMA PET in improving metastatic disease detection. Of note, 18F-PSMA PET (Pylarify), approved in 2021 after 68Ga-PSMA PET in 2020, may identify lesions with a higher standardized uptake value (SUV) as well as detect disease sites with less intense focus [37,38]. This paves the way for other PSMA-based radiotracers in development, such as Flotufolastat F18 [39].

Accumulating data have shown that the number of PSMA PET-positive sites for patients with hormone-sensitive prostate cancer (HSPC) can be highly prognostic. In a retrospective analysis of 450 men with biochemically recurrent prostate cancer post-prostatectomy, Schweizer et al. found that patients with fewer than three metastatic sites had improved survival compared to those with greater than three metastatic lesions (HR 0.50; 95% CI, 0.29–0.85) [40]. In another retrospective analysis of 369 prostate cancer patients treated with external beam radiotherapy (EBRT), patients with less than or equal to five metastatic sites had significantly better survival rates than patients with greater than five metastatic sites [41].

Early retrospective evidence suggests that PSMA PET has led to earlier and more aggressive clinical management for patients with BCD [42]. In a retrospective analysis, starting treatment based on PSMA PET positivity appeared to improve progression-free survival [42]. Although earlier treatment is an attractive approach, it is important to consider the negative quality of life and associated cumulative complications when starting ADT. As such, early evidence is hypothesis-generating and requires further validation; it also represents an opportunity to study both escalation and de-escalation approaches. Further studies exploring optimal treatment strategies for OMPC are necessary and currently underway.

## 4. Current Treatment Strategies

*Cytoreduction: Targeting the Primary Tumor:* Preclinical studies have demonstrated that cytoreductive radiation or surgical removal of the prostate in the setting of metastatic disease leads to a decrease in CTCs and disseminated tumor cells (DTCs) [43]. These cells would otherwise both seed new metastases and lead to the remodeling of the pre-metastatic niche [43]. In a rat xenograft model of metastatic prostate cancer, cytoreductive surgery was found to significantly reduce metastatic burden [44]. Namely, cytoreductive surgery has been found to enhance the immune response by increasing the ratio of T helper cells to regulatory T cells in the blood, thus permitting the anti-tumor effect of certain intratumoral immunotherapies [44]. These findings serve as the basis for cytoreduction in the treatment of OMPC.

Abscopal Effect: The abscopal effect is a well-recognized phenomenon in which radiation therapy targeting the primary tumor or metastatic sites decreases the growth of non-irradiated tumors that are distant from the radiation field [26]. This effect is believed to be immune-mediated, as cells killed by ionizing radiation are thought to produce tumor-specific antigens, changes in immune cells in peripheral blood, increases in antibody responses to other antigens, and stimuli that lead to the maturation of dendritic cells [45]. These inflammatory signals produced by ionizing radiation lead to the activation of tumor-specific T cells. Demaria et al. found evidence in support of this hypothesis using mice with syngeneic mammary carcinomas. They found that radiotherapy (RT) with a dose of 2 Gy or 6 Gy delivered in a single fraction using a cobalt 60 source reduced the growth of non-irradiated tumors; however, this effect was tumor-specific, as certain tumors like lymphomas did not respond in this manner [26]. These findings create the framework of the reasoning behind the targeted treatment of the primary and metastatic tumors in OMPC, as it is believed that both the metastatic lesions and the primary tumor itself lead to molecular signaling that promotes further disease progression.

Cytoreductive Prostatectomy: Using the Surveillance Epidemiology and End Results (SEER) database, Culp et al. found that among the 8185 identified patients diagnosed with stage IV prostate cancer, those who underwent radical prostatectomy or prostate brachytherapy were found to have a significantly higher 5-year overall survival and predicted disease-specific survival than those who did not undergo any definitive treatment of the prostate [46]. Gratzke et al. performed a similar analysis using the Munich Cancer Registry and also found a significant survival benefit for radical prostatectomy in patients with newly diagnosed metastatic prostate cancer [47]. This approach was found to be relatively safe in this patient population, with an acceptable complication rate in select patients with locally resectable, distant metastatic prostate cancer [48].

Heidenreich et al. evaluated functional and oncological outcomes in men with low-volume metastatic prostate cancer who had undergone cytoreductive prostatectomy (CRP), and in this retrospective, multi-institutional study, CRP was associated with a 5-year overall survival of 80% [49]. Importantly, there was a significant association between neoadjuvant ADT, low-volume disease, and preoperative PSA with regard to a lower risk of surgery-related complications, suggesting that CRP could be a viable treatment option in such patients [49]. Steuber et al. prospectively compared 43 patients with low-volume bone metastases who underwent CRP to a control group of 40 patients who received the best systemic therapy (BST). In this non-randomized study, a survival benefit was not observed, and patients who underwent CRP had significantly lower locoregional complications [50]. Of note, patients who underwent CRP were younger, had lower CT stage, lower PSA at diagnosis, and fewer bone metastases than patients in the control group who received BST.

Cumulatively, these retrospective studies suggest that CRP is a feasible and tolerable approach with a palliative benefit in select patients with OMPC [50]. Currently, there are several prospective studies evaluating the role of CRP in OMPC patients. NCT02971358 is a single-center, phase I/II study evaluating the rate of perioperative complications within 90 days of CRP in patients with OMPC or very high-risk locally advanced prostate cancer. NCT04992026 is a phase II randomized, controlled, multicenter study evaluating the safety and efficacy of CRP in hormone-sensitive OMPC patients receiving abiraterone plus ADT with or without CRP. Furthermore, NCT03298087 and NCT05212857 are ongoing studies evaluating the combination of CRP, systemic therapy, and MDT with SBRT in OMPC patients. The findings of these prospective studies have the potential to provide clinical validation for implementing CRP as SOC for select OMPC patients moving forward.

Prostate Radiotherapy: Cumulating evidence that radiation and cytoreduction can be beneficial in patients with metastatic prostate cancer has led to further investigation into this approach. Using the National Cancer Database (NCDB), Rusthoven et al. demonstrated that ADT plus prostate RT in 6382 men with metastatic prostate cancer was associated with an improved OS compared to ADT alone. A secondary analysis revealed that both RT and prostatectomy combined with ADT were similarly superior to ADT alone [51]. A similar retrospective study by Cho et al. found that RT to the primary tumor improved OS and biochemical failure-free survival in metastatic prostate cancer patients [52]. Of note, stratified analysis demonstrated this survival benefit to be greater in patients with good performance status and only bone metastases at diagnosis [52].

Metastasis-Directed Therapy: While metastasis-directed therapy (MDT) has historically been used for symptom palliation, there is mounting evidence that this approach may indeed improve cancer outcomes. In fact, in 2022, the European Society for Radiotherapy and Oncology (ESTRO) Guidelines Committee published that MDT should be recommended in de novo oligometastatic, oligorecurrent, and oligo-progressive disease in patients with bone, visceral, and nodal metastases [16].

A meta-analysis of 7 studies by Ost et al. on patients with metastatic prostate cancer treated with either surgical metastectomy or non-palliative radiotherapy found that 51% of patients were progression-free at 1–3 years post-MDT [53]. In a separate multi-institutional pooled cohort, Ost et al. retrospectively studied patients who underwent MDT with stereotactic body radiotherapy (SBRT) in oligorecurrent disease [54]. SBRT was generally safe and demonstrated prolonged progression-free survival in a subset of patients with limited metastatic recurrence after treatment of their primary tumor [54]. In a multi-institutional, retrospective, matched-case analysis of men with nodal oligorecurrent prostate cancer, Steuber et al. compared ADT to MDT with salvage lymph node dissection (SLND) or SBRT at PSA progression. With a 5-year cancer-specific survival of 95.7% (95% CI, 93.2–97.3) versus 98.6% (95% CI, 94.3–99.6, *p* = 0.005), this study suggests MDT may be beneficial in managing nodal oligorecurrent disease [55]. While this study did not compare SLND to SBRT, these treatments are used interchangeably with comparable results [54,56].

A recent retrospective analysis by Lanfranchi et al. evaluated the impact of radiotracer selection on oncological outcomes in OMPC patients treated with PET/CT-guided MDT. Out of 37 patients with no more than 5 metastatic lesions, 11 received [18F]F-Flurocholine-guided MDT and 26 received [68Ga]Ga-PSMA-11-guided MDT. [68Ga]Ga-PSMA-11-guided MDT was found to be associated with improved progression-free survival, suggesting a favorable oncological impact of using this radiotracer to guide MDT in OMPC [57]. It is possible that, eventually, 18F-PSMA (Pylarify) PET may supersede the ability of [68Ga]Ga-PSMA in identifying and treating metastases [37,38]. In another systematic review of 56 studies on the use of MDT in PSMA PET-avid OMPC patients, Rogowski et al. found that MDT with radiotherapy was associated with high local control rates and improved progression-free survival. Notably, these findings were consistent amongst both hormone-sensitive and castration-resistant diseases [58].

These findings paved the way for several prospective studies summarized in Table 1. In the phase I trial POPSTAR, 33 men with oligorecurrent prostate cancer and no more than 3 metastases of bone or lymph nodes received a single fraction of stereotactic ablative radiotherapy (SABR) to all visible metastatic sites of disease. The treatment was feasible in most (97%), and impressively, two-year freedom from ADT was 48% (95% CI, 31–75) in the 22 patients who were castration-sensitive [59]. In the randomized phase II trial SABR-COMET, 99 men were randomized to either SOC systemic therapy or SOC systemic therapy plus SBRT to up to 5 metastatic sites [60]. The combination arm was found to have superior 5-year overall survival (OS) (42.3% vs. 17.7%, *p* = 0.006). While the quality of life scores were comparable, the combination group did experience more grade ≥ 2 adverse events (29% vs. 9%) [60]. In another phase II randomized trial of STOMP, asymptomatic men with oligorecurrent hormone-sensitive prostate cancer (≤3 metastatic sites) underwent either surveillance or received MDT to all metastatic sites with surgery or SBRT [61]. Five-year ADT-free survival (34% vs. 8%; HR 0.57, *p* = 0.06) and castration-resistant prostate cancer (CRPC)-free survival (76% vs. 53%; HR 0.62, *p* = 0.27) favored those who received MDT [62]. Additionally, the ORIOLE study randomized 54 men with oligorecurrent HSPC and no more than 3 metastatic sites in a 2:1 ratio to receive SABR versus observation. The SABR group had less progression at 6 months (19% vs. 61%), and importantly, radiotherapy was associated with a systemic immune response, as T-cell receptor sequencing demonstrated significantly increased clonotypic expansion [63]. Furthermore, circulating tumor DNA (ctDNA) analysis demonstrated a significantly lower ctDNA concentration in OMPC patients compared to more advanced metastatic CRPC (mCRPC) and metastatic HSPC (mHSPC) patients [63]_._ ctDNA analysis was also used to identify a high-risk mutation signature that may help distinguish patients who would not benefit from MDT with SABR [63]_._ Clinical benefit was, however, observed in patients with a greater baseline T-cell clonality. The findings of these studies provide proof of the principle that MDT with radiotherapy is safe and may be clinically beneficial in select patients [63].

The role of MDT for men with PSMA PET-avid-only disease was investigated in PSMA-MRgRT, a single-institution study with rising PSA after maximal local therapy for patients with less than 5 metastatic sites as determined by PSMA-PET-MR/CT [64]. This was a single-institution, single-arm study in which 37 of the enrolled patients agreed to receive ADT and MDT with either SABR or surgery. The overall response rate was 60%, and 22% had biochemical “no evidence of disease”, defined as a 100% PSA decline from baseline [64]. More recently, See et al. reported a five-year follow-up of TRANSFORM, a prospective clinical trial investigating the impact of MDT with SBRT on treatment escalation-free survival (TE-FS) in 199 OMPC patients treated with SBRT to metastatic sites [65]. The rate of TE-FS—freedom from further treatment beyond SBRT—was 21.7% (95% CI, 15.7–28.7%) after five years, suggesting that systemic treatment escalation may be effectively delayed in some OMPC patients treated with SBRT-based MDT [65].

Combining MDT with ADT: The phase I OLIGOPELVIS GETUG P07 trial treated 67 men with oligorecurrent pelvic node-only relapse (≤6 metastatic pelvic lymph nodes) with high-dose intensity-modulated salvage pelvic RT and 6-month-long ADT, with a promising median PFS (45.3 months) and median biochemical recurrence-free survival (25.9 months), with 46% being relapse-free at 3 years [66]. EXTEND was a phase II basket randomized study including multiple solid tumors, which evaluated the effect of combining MDT with SOC systemic therapy. Eighty-seven men with OMPC (≤5 sites of metastasis) were randomized to receive either ADT or MDT with definitive RT to all metastatic sites combined with intermittent ADT [67]. Patients who received the combination were found to have superior progression-free survival (PFS), and in their blood samples, there was a greater immune response with an increased proportion of CD4^+^ and CD8^+^ T-cells with high and intermediate Ki-67 and programmed cell death protein 1 expression [67]. In addition, expansion and remodeling of peripheral CD8^+^ T-cell receptor repertoire, which reflects intratumoral changes, were also observed [67]. Thus, combining MDT with ADT may lead to improved oncological outcomes, potentially in part due to heightened immune stimulation [67].

### 4.1. Combination Systemic Therapy Alone

Currently, the American Society of Clinical Oncology (ASCO) guidelines recommend either doublet or triplet systemic therapy with ADT combined with docetaxel, abiraterone, enzalutamide, apalutamide, or darolutamide as SOC for mHSPC, although there is little guidance on optimal patient selection and treatment sequence [68]. While it appears that the addition of docetaxel as a doublet combination (CHAARTED) is most beneficial in those with a high burden of disease (at least four sites of bone metastases with at least one outside the vertebral column or pelvis and/or visceral metastasis), the OS benefit as triplet therapy in STAMPEDE with docetaxel persists regardless of metastatic burden [69,70].

ARASENS is a phase III randomized study with mHSPC men treated with ADT and docetaxel ± darolutamide, in which the triplet combination demonstrated superior OS and time to castration resistance. However, to date, subgroup analysis based on disease volume/risk has not been reported [71], thus limiting insight for those with OMPC. PEACE-1 is another randomized phase II study previously mentioned that similarly has not yet published outcomes data based on disease burden and risk. Subgroup analyses from PEACE-1 and STAMPEDE both suggest that radiation of the primary tumor is safe and effective in patients with low-volume de novo mHSPC, with a comparable toxicity profile and quality of life with the addition of radiotherapy [12,72]. Ongoing studies evaluate the use of escalation and de-escalation strategies based on clinical criteria.

One clinical dilemma is determining which androgen receptor (AR) signaling-targeting agent to select for patients with OMPC. Long-term follow-up from ARCHES, a randomized phase 3 study of ADT ± enzalutamide in men with mHSPC, revealed that adding enzalutamide to ADT significantly reduced the risk of metastatic progression and emergence of CRPC even in patients with low-volume disease [73]. To address this, Wenzel et al. conducted a network meta-analysis on 7 randomized phase III trials comparing combination therapy with ADT plus enzalutamide, docetaxel, abiraterone, or apalutamide to ADT alone in mHSPC patients. After stratification between low- and high-volume mHSPC, they found that when compared to ADT alone, only abiraterone and enzalutamide resulted in improved OS in the low-volume subgroup, with HR 0.61 (95% CI, 0.47–0.79) and 0.43 (95% CI, 0.26–0.72), respectively [74]. Furthermore, in another systematic review and network meta-analysis comparing these combination therapies in mHSPC patients, Menges et al. found that the benefit-harm balance was more favorable in hormonal combination treatments than in combination treatments involving chemotherapy [71,75]. A real-world study by Morgans et al. concluded that darolutamide may be the best androgen receptor-targeting agent (ARTA) doublet option in patients with non-metastatic CRPC (nmCRPC), a clinically distinct state of disease. Lastly, as ARTA may increase severe adverse effects such as cardiovascular complications, one should take such factors into consideration when making such a decision [76,77].

*Concurrent Combination Systemic Therapy with Prostate Cytoreduction:* Aggressive multimodality, using combination systemic therapy with prostate radiotherapy, could potentially further improve clinical outcomes in patients with synchronous OMPC. In STAMPEDE, a multicenter, multi-arm randomized control trial in the UK that enrolled 2061 men, researchers sought to better understand the impact of adding prostate radiotherapy to SOC systemic treatment for men with metastatic prostate cancer. While the study did not meet its primary endpoint, in the cohort of men harboring low-burden metastatic disease (40%), radiotherapy did indeed improve OS (73% vs. 81% at 3 years) in subgroup analysis [12]. The HORRAD trial compared ADT to ADT plus RT in patients with primary bone metastatic prostate cancer and found no statistically significant survival benefit for RT for the overall population [78]. However, the time to PSA progression was 15 months (95% CI, 11.8–18.2) in the RT group compared with 12 months (95% CI, 10.6–13.4) in the ADT-only group [78]. Additionally, in the randomized phase III 4-arm PEACE-1 study, where men with de novo metastatic prostate cancer received ADT + abiraterone ± docetaxel ± prostate radiotherapy, prostate irradiation combined with systemic therapy did not improve overall survival); those who received the “quadruple” combination did have the best radiographic PFS [72]. While further prospective validation is needed, these studies provide proof of principle that combining prostate radiotherapy with standard systemic therapy may be an attractive approach in patients with OMPC.

**Figure 2:**
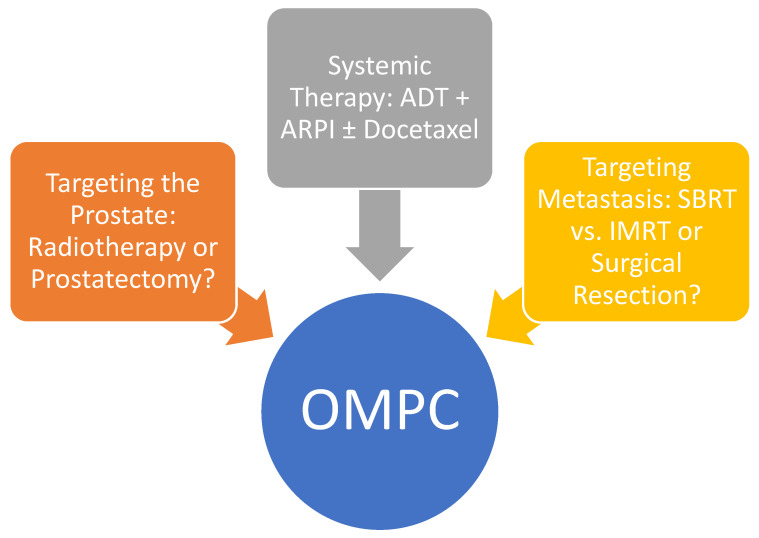
Treatment strategies for OMPC. Abbreviation: ARPI = Androgen receptor pathway inhibitor; ADT = Androgen deprivation therapy; IMRT = Intensity-modulated radiotherapy; OMPC = Oligometastatic prostate cancer; SBRT = Stereotactic body radiotherapy.

The current SOC for the treatment of oligometastatic HSPC is combination systemic therapy, but there are cumulating data that multimodality approaches, including primary prostate RT and MDT, can improve outcomes for some patients (Figure 2). SOLAR is a prospective phase II single-arm study investigating a multimodal therapeutic approach combining systemic therapy, MDT, and primary tumor-directed therapy in OMPC patients [79]. Prostate cancer patients with one to five visible metastases on PET/CT and no prior treatment underwent primary tumor-directed therapy with either RP or RT, systemic therapy with up to six months of leuprolide, apalutamide, and abiraterone acetate plus prednisone, and MDT with SBRT [79]. The primary endpoint of testosterone recovery ≥ 150 ng/dL and undetectable serum PSA for RP or <2 ng/mL for RT at 6 months after recovery was achieved in 20/24 (83%) patients. In addition, all patients were progression-free at a median of 31 months (range 19.4–62 months) [79]. These findings suggest that an aggressive multimodal therapeutic approach is feasible and promising, although prospective randomized validation is needed [79]. Given the poor quality of life associated with long-term ADT, there has been growing interest in finding alternative therapies that can replace or delay the initiation of androgen suppression [80]. Ongoing investigation will continue to define this rapidly evolving field.

### 4.2. Castration-Resistant OMPC: Can We Take the Same Approach?

Metastatic CRPC (mCRPC) is the lethal phenotype of prostate cancer, with a median survival of 14 months (range 9–30) after the development of castration resistance [81]. Few studies have been reported to better understand the optimal treatment approach for oligometastatic CRPC; however, patients with low metastatic burden have generally been found to have better outcomes among mCRPC patients [82,83]. This provides an opportunity to investigate alternative therapeutic approaches that best balance treatment efficacy with potential side effect profiles. The site of metastasis, rather than the burden of disease, is used for prognosis evaluation for patients with mCRPC [84]. Systemic treatment with sipuleucel-T in patients who are asymptomatic or minimally symptomatic has demonstrated improved OS in patients with low-volume mCRPC [85,86,87]. However, limited data exist to help select systemic treatment strategies based on disease volume.

Zhang et al. conducted a prospective phase II clinical trial evaluating the effect of SABR on clinical outcomes and immunological response in 89 patients with 11C-Choline-PET/CT-identified oligometastatic CRPC [88]. OS in these patients was 96% and 80% at 1- and 2-year follow-up, respectively. Elevated levels of tumor-reactive effector T-cells (T_TR_) at baseline were associated with improved PFS [88]. Patients who exhibited an increase in T_TR_ 14 days after baseline had improved OS, suggesting that RT leads to T-cell changes that result in a survival advantage [88] and that MDT may be effective in a subpopulation of patients with oligometastatic CRPC. The promise of SABR in oligometastatic CRPC was also observed in a prospective study by Francolini et al., in which 157 OMPC patients received abiraterone acetate and prednisone and were treated with or without the addition of SABR [89]. The rate of biochemical response, defined as PSA decrease ≥ 50% from baseline six months after treatment initiation, was 92% in the abiraterone and prednisone + SABR arm vs. 68.3% in the control arm, which received abiraterone and prednisone alone, with an odds ratio (OR) of 5.34 (2.05 to 12.88; *p* = 0.001) in favor of the addition of SABR [89].

## 5. Future Directions

With the rapid development of better diagnostic and therapeutic modalities, it is reasonable to hypothesize that outcomes will continue to improve for patients with OMPC. The pace of advancement will be critically dependent on three important pillars: diagnostic accuracy, novel therapeutic strategies, and application of artificial intelligence.

### 5.1. Diagnostic Accuracy

Advances in diagnostic accuracy, such as the use of PSMA-based PET imaging, have resulted in the earlier diagnosis of patients with OMPC. Other radiotracers currently in development, including gastrin-releasing peptide receptors and Ga-fibroblast activation protein inhibitors, have shown early promise [90]. As better imaging becomes available, increased diagnostic sensitivity and specificity of such imaging can improve disease characterization and, importantly, guide clinicians to select the optimal treatment approach.

Second, next-generation sequencing (NGS) technologies have been increasingly utilized in recent years to inform the molecular characterization of tumors and their heterogeneity [91]. For example, loss of the PTEN tumor suppressor gene on chromosome 10 and loss of TP53 are common secondary genetic events associated with metastasis, disease progression, and higher tumor stage [28,92,93,94]. Furthermore, mutations in BRCA1/2 are associated with poorer outcomes in men with prostate cancer. Although treatment with poly-ADP ribose polymerase inhibitors has demonstrated definitive clinical benefit for men with BRCA1/2 + mCRPC [95], it is less clear at this time whether those with OPMC will benefit similarly to those with widely metastatic disease, as further clinical validation will be warranted. Additionally, gene expression profiling of the primary prostate tumor in those with meta-synchronous OMPC may help guide treatment decisions. Several commercially available platforms, such as Decipher and Oncotype Dx, have demonstrated prognostic value in patients with localized disease based on CT and bone scans. It is possible that some patients within this population may harbor micro-metastatic or PSMA PET + disease, and further investigation in assessing the prognostic and predictive value of these genomic panels will be needed.

Third, blood-based biomarkers may offer additional opportunities to improve outcomes in OMPC. Loyfer et al. developed a DNA methylation atlas with 39 normal cell types with 25 unmethylated markers each [96]. Further investigation into these markers and their relationship with known prostate-cancer-specific hypermethylated markers could lead to a greater understanding of lineage-plasticity-specific markers involved in disease progression. Another opportunity is the use of ctDNA, as quantitative ctDNA has been shown to be independently reliable in prognosticating and predicting therapeutic response and overall survival [97]. Future prospective, randomized studies should prioritize NGS testing and liquid biopsy in order to better understand the optimal utilization of available treatment approaches. The development of such noninvasive blood-based tests to best characterize disease states can guide treatment selection (for example, MDT vs. systemic treatment) for OMPC, leading to improved outcomes [28].

### 5.2. Novel Therapeutic Strategies

There are several novel therapeutic approaches in the clinical development of the treatment of metastatic prostate cancer: inhibition of androgen receptor signaling, immune-based treatments, antibody–drug conjugates, epigenetic therapies, and targeting radioligand emitters, among others (see Table 2). As these novel therapies enter randomized prospective clinical evaluation, their relative benefit for patients with OMPC will need to be carefully assessed. In addition, several ongoing clinical trials are investigating concurrent multimodality treatment for OMPC (Table 2). START-MET, for example, is a phase III randomized study in which patients receive SOC treatment with ADT, RT to the primary tumor, and second-generation hormonal therapy with or without SBRT to all metastatic lesions. METRO is another ongoing randomized phase III clinical trial comparing SOC with ADT and local RT for de novo patients to SOC plus SBRT to all PSMA-positive lesions. Along with other similarly designed trials in progress, together these studies may provide clinical validation of whether “more is better” for patients with OMPC.

### 5.3. Artificial Intelligence

Deep machine learning, also known as artificial intelligence (AI), can more effectively combine key data from a large number of different sources to allow for a better-personalized approach. A recent study by Armstrong et al. demonstrated the validity of an AI-derived clinical and histopathological predictive biomarker of long-term ADT benefit with RT in localized high-risk prostate cancer patients [98]. Ultimately, improving outcomes in patients with OMPC will require analysis of individual patient records (e.g., electronic medical records), diagnostic testing results (e.g., PSMA PET imaging), specialized testing (e.g., biomarker profiling), and a large number of patient data sets so we can better understand the optimal strategy [99].

## 6. Conclusions

OMPC is a poorly defined heterogeneous clinical state but also offers an opportunity for the development of more effective treatment approaches going forward. There is consensus that OPMC should be defined as less than five sites of non-visceral metastasis, which should be targetable via surgical resection or radiotherapy, but inclusion based on the site of disease (e.g., lung), the timing of metastatic disease (de novo versus recurrence), and how to best incorporate a targeted approach (prostate or metastatic site) remains undefined. Several evolving diagnostic modalities may more precisely help us better define OMPC, although none has been validated to date and is commercially available. Optimal therapeutic strategy for OMPC has rapidly evolved, with a focus on intensification and de-intensification of systemic therapy, utilization of prostate and metastasis-directed therapy, and development of novel biomarkers to better guide a multimodality approach. Continued development of novel therapeutic strategies, improving diagnostic accuracy, and utilization of artificial intelligence tools will be key in driving future advancement, and ongoing clinical trials will continue to shape the optimal treatment strategy moving forward.

## Figures and Tables

**Table 1:** Prospective Studies Utilizing MDT For Men with OMPC.

NCT	Acronym	Phase	Treatment	Primary Outcome
01446744	SABR-COMET	2	SBRT + SOC ST	OS
	POPSTAR	1	SBRT	ADT-FS
01558427	STOMP	2	Salvage (surgical or radiotherapy) of metastasis	ADT-FS
02680587	ORIOLE	2	SBRT	Progression at 6 months
03784755	OLIGOPELVIS GETUG P07	2	High dose IMRT, Eligard	Biochemical or clinical relapse free survival (2y)
	SPMA-MRgRT	2	(18F) DCFPyL, PET/MRI	Biochemical response
03599765	EXTEND	2	RT to all metastatic sites + hormonal therapy	Disease progression

Abbreviation: ADT-FS = Androgen deprivation therapy-free survival; IMRT = Intensity-modulated radiotherapy; MDT = Metastasis-directed therapy; OMPC = Oligometastatic prostate cancer; OS = Overall survival; RT = Radiotherapy; SBRT = Stereotactic body radiotherapy; SOC ST = Standard of care systemic therapy.

**Table 2:** Ongoing Clinical Trials for Treatment of OMPC.

NCT	Phase	Enrollment	Experimental	Control	Primary Outcome
NCT04992026	II	128	S + ADT + abi	ADT + abi	TT PSA progression TT Radiographic progression
NCT02971358	I/II	200	RP	None	The rate of complications 90 days after S
NCT04610372 (PROMPT)	N/A	168	Br or SABR	5500 cGy	Urinary symptoms
NCT05183074	II	50	MR—linac SBRT	None	The Incidence of Acute GU and GI toxicities
NCT04190446	II	83	PBRT or IMRT	None	Proportion of pts with >/=grade 3 GI and/or GU toxicity
NCT05223803	II	122	SABR + BST + PPRT	BST + PPRT	2-year FFS
NCT02563691 (CROP)	I/II	90	SABR	None	Incidence of late RT toxicities
NCT03556904	II	13	Ablative Radiation + SOC	SOC	mDOR
NCT04037358 (RAVENS)	II	64	Radium-223 + SABR	SABR	PFS
NCT04115007 (PRESTO)	III	350	SBRT + SOC	SOC	CRPC free survival
NCT04983095 (METRO)	III	114	SBRT + SOC	SOC	FFS
NCT04619069	I/II	30	SBRT + SOC	SOC	Proportion of eligible patients
NCT03304418 (RROPE)	II	20	Radium-223 + RT	None	Time to ADT use
NCT04300673 (DETECT)	I/II	20	Radio-guided surgery	None	Feasibility of 111IN-PSMA-1&T radio-guided surgery
NCT04141709 (OLI-CR-P)	II	66	Local ablative radiotherapy	Observation	Time to PSA progression
NCT04599686	N/A	100	SBRT	ADT	1-year ADT-free survival
NCT03630666 (OLIGOPELVIS2)	III	256	Radiotherapy + IADT	IADT	PFS
NCT05209243 (START-MET)	III	266	SBRT + SOC	SOC	rPFS
NCT05079698	I	6	SBRT + 177Lu-PSMA-617	None	Proportion of subjects with dose limiting toxicity
NCT01558427	II	62	S or RT to metastases	AS	ADT free survival
NCT03525288 (PSMA-PETgRT)	II/III	130	PSMA-PETgRT	SOC RT	FFS
NCT03569241 (STORM)	II	196	MDT + WPRT + ADT	MDT + ADT	MFS
NCT05053152 NRG PROMETHEAN	II	260	Relugolix + SABR	Placebo + SABR	rPFS
NCT03940235 RADIOSA	II	150	SBRT + ADT	SBRT	PFS
NCT05404139	II	66	Enzalutamide + SBRT + ADT	SBRT + ADT	PFS
NCT03503344 PILLAR	II	60	Apalutamide + SBRT	SBRT	Proportion of patients with undetectable PSA
NCT05352178 SPARKLE	III	873	MDT + 1 month ADT or MDT + 6 months ADT + enzalutamide	MDT	Polymetastatic free survival
NCT04641078 (DART)	II	124	Darolutamide + SBRT	SBRT	MFS
NCT04748042	II	29	Abiraterone + ADT + radiation + olaparib	None	% pts without treatment failure at 24 months
NCT02274779 OLIGOPELVIS	II	70	IMRT + ELIGARD	None	Biochemical or clinical relapse-free survival (2 years)
NCT03902951	II	28	Leuprolide + apalutamide + abiraterone +SBRT	None	% patients achieving a serum PSA of <0.05 ng/mL
NCT04175431	II	100	Fluciclovine PET/CT + abiraterone + prednisone	Fluciclovine PET/CT	Undetectable PSA (<0.2 ng/mL) rate
NCT00544830	II	29	ADT + RT	None	TT PSA Relapse
NCT03361735	II	24	Leuprolide acetate or goserelin acetate + SBRT + Ra 223	None	1. TT treatment failure 2. ORR
NCT03298087	II	28	Prostatectomy + SBRT + Leuprolide + apalutamide + abiraterone	None	PSA < 0.05 ng/mL (RP) or PSA <nadir + 2 ng/mL (PRBT)
NCT05212857	II	160	Systemic treatment + prostatectomy + SBRT	None	rPFS at 2 years
NCT05496959 (LUNAR)	II	100	177Lu-PNT2002 IV + SBRT	SBRT	PSMA PET/CT-based PFS
NCT04011410	II	20	Hydroxychloroquine	None	PAR-4 Level
NCT04443062 (BULLSEYE)	II	58	177Lu-PSMA radioligand therapy	SOC	Disease Progression (EOT1)
NCT05146973	II	50	177Lu-DOTA-TLX591	None	PSA PFS
NCT03007732	II	23	SD-101 + ADT + SBRT + Pembrolizumab	ADT + SBRT + Pembrolizumab	Change Rate of PSA < nadir + 2 ng/mL (up to 15 months)
NCT03795207 (POSTCARD)	II	96	SBRT + DURVALUMAB	SBRT	Two-years PFS

Ab = Abiraterone; ADT = Androgen deprivation therapy; AS = Active surveillance; Br = Brachytherapy; BST = Best supportive therapy; CRPC = Castration-resistant prostate cancer; EOT1 = End of study period treatment; FFS = Failure-free survival; IADT = Intermittent androgen deprivation therapy; IMRT = Intensity-modulated radiotherapy; mDOR = Median duration of response; MDT = Metastasis-directed therapy; MFS = Metastatic-free survival; ORR = Objective response rate; PPRT = Primary prostate radiotherapy; PRBT = Prostatic bed radiotherapy; Pts = Patients; RP = Radical prostatectomy; rPFS = radiographic progression-free survival; RT = radiotherapy; S = Surgery; SABR = Stereotactic ablative radiotherapy; SOC = Standard of care; TT = Time to; WPRT = Whole prostate radiotherapy.

## Abbreviations

ADT	androgen deprivation therapy
AI	artificial intelligence
AR	androgen receptor
ARTA	androgen receptor targeting agent
ASCO	American Society of Clinical Oncology
BCD	biochemically recurrent disease
BST	best systemic therapy
cfDNA	cell-free DNA
CI	confidence interval
CRP	cytoreductive prostatectomy
CRPC	castration-resistant prostate cancer
CT	computed tomography
CTCs	circulating tumor cells
ctDNA	circulating tumor DNA
DDSB	DNA double-strand break
DTCs	disseminated tumor cells
EBRT	external beam radiotherapy
EMT	epithelial–mesenchymal transition
ESTRO	European Society for Radiotherapy and Oncology
HR	hazard ratio
HSPC	hormone sensitive prostate cancer
mCRPC	metastatic castration-resistant prostate cancer
MDT	metastasis directed therapy
mHSPC	metastatic hormone-sensitive prostate cancer
MMR	mismatch repair
MRI	magnetic resonance imaging
MSI-H	microsatellite instability high
NCDB	National Cancer Database
NGS	next-generation sequencing
nmCRPC	non-metastatic castration resistant prostate cancer
OM	oligometastatic
OMPC	oligometastatic prostate cancer
OR	odds ratio
OS	overall survival
PET	Positron Emission Tomography
PFS	progression-free survival
PSMA	prostate-specific membrane antigen
PTEN	phosphatase and tensin homolog
rPFS	radiographic progression-free survival
RIPK2	receptor-interacting protein kinase 2
RP	radical prostatectomy
RT	radiotherapy
SABR	stereotactive ablative radiotherapy
SBRT	stereotactic body radiotherapy
SEER	Surveillance Epidemiology and End Results
SLND	salvage lymph node dissection
SOC	standard of care
STAMPEDE	Systemic Therapy in Advancing or Metastatic Prostate Cancer: Evaluation of Drug Efficacy
SUV	standardized uptake value
Treatment escalation-free survival	TE-FS
T_TR_	tumor reactive effector T-cells
VEGFR1	vascular endothelial growth factor receptor 1
